# A Robust, Safe, and Scalable Magnetic Nanoparticle Workflow for RNA Extraction of Pathogens from Clinical and Wastewater Samples

**DOI:** 10.1002/gch2.202000068

**Published:** 2021-02-22

**Authors:** Gerardo Ramos‐Mandujano, Rahul Salunke, Sara Mfarrej, Andri Taruna Rachmadi, Sharif Hala, Jinna Xu, Fadwa S. Alofi, Asim Khogeer, Anwar M. Hashem, Naif A. M. Almontashiri, Afrah Alsomali, Digambar B. Shinde, Samir Hamdan, Pei‐Ying Hong, Arnab Pain, Mo Li

**Affiliations:** ^1^ Biological and Environmental Sciences and Engineering Division (BESE) King Abdullah University of Science and Technology (KAUST) Thuwal 23955‐6900 Kingdom of Saudi Arabia; ^2^ King Abdullah International Medical Research Centre King Saud University for Health Sciences Ministry of National Guard Health Affairs Jeddah 21859 Saudi Arabia; ^3^ Infectious Diseases Department King Fahad Hospital Almadinah Almunwarah 11525 Saudi Arabia; ^4^ Plan and Research Department General Directorate of Health Affairs Makkah Region Ministry of Health Mecca 11176 Saudi Arabia; ^5^ Vaccines and Immunotherapy Unit King Fahd Medical Research Center King Abdulaziz University Jeddah 21859 Saudi Arabia; ^6^ Department of Medical Microbiology and Parasitology Faculty of Medicine King Abdulaziz University Jeddah 21859 Saudi Arabia; ^7^ College of Applied Medical Sciences Taibah University Almadinah Almunwarah 71491 Saudi Arabia; ^8^ Center for Genetics and Inherited Diseases Taibah University Almadinah Almunwarah 71491 Saudi Arabia; ^9^ Infectious Diseases Department King Abdullah Medical Complex Jeddah 24246 Saudi Arabia; ^10^ Division of Physical Science and Engineering King Abdullah University of Science and Technology (KAUST) Thuwal 23955‐6900 Kingdom of Saudi Arabia

**Keywords:** influenza, magnetic nanoparticles, nucleic acid purification, SARS‐CoV‐2, wastewater surveillance

## Abstract

Molecular diagnosis and surveillance of pathogens such as SARS‐CoV‐2 depend on nucleic acid isolation. Pandemics at the scale of COVID‐19 can cause a global shortage of proprietary commercial reagents and BSL‐2 laboratories to safely perform testing. Therefore, alternative solutions are urgently needed to address these challenges. An open‐source method, magnetic‐nanoparticle‐aided viral RNA isolation from contagious samples (MAVRICS), built upon readily available reagents, and easily assembled in any basically equipped laboratory, is thus developed. The performance of MAVRICS is evaluated using validated pathogen detection assays and real‐world and contrived samples. Unlike conventional methods, MAVRICS works directly in samples inactivated in phenol‐chloroform (e.g., TRIzol), thus allowing infectious samples to be handled safely without biocontainment facilities. MAVRICS allows wastewater biomass immobilized on membranes to be directly inactivated and lysed in TRIzol followed by RNA extraction by magnetic nanoparticles, thereby greatly reducing biohazard risk and simplifying processing procedures. Using 39 COVID‐19 patient samples and two wastewater samples, it is shown that MAVRICS rivals commercial kits in detection of SARS‐CoV‐2, influenza viruses, and respiratory syncytial virus. Therefore, MAVRICS is safe, fast, and scalable. It is field‐deployable with minimal equipment requirements and could become an enabling technology for widespread testing and wastewater monitoring of diverse pathogens.

## Graphical abstract

One important bottleneck in the diagnosis and surveillance of COVID‐19 is the shortage of kits for RNA extraction. Magnetic‐nanoparticle‐aided viral RNA isolation from contagious samples (MAVRICS) is an open‐source, safe, fast, and scalable method for RNA extraction. MAVRICS rivals commercial kits but requires minimal materials, and thus could become an enabling technology for widespread community testing of diverse pathogens.

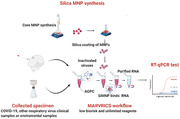

## 1 Introduction

Testing for COVID‐19 is vital for monitoring and mitigating the spread of SARS‐CoV‐2 and for safely restarting the normal economy. To date, molecular diagnosis of COVID‐19 predominantly relies on detection of SARS‐CoV‐2 RNA using real‐time reverse transcription polymerase chain reaction (rRT‐PCR) assays, such as those approved by the US Centers for Disease Control and Prevention (CDC).^[^ ^1^ ^]^ As SARS‐CoV‐2 spreads globally, it also accumulates approximately 1 to 2 single nucleotide variants (SNVs) in the 29 903 bp genome per month.^[^ ^2^ ^]^ The emergence of new strains could have serious implications in the efficacy of diagnostic tests and success of vaccines. For example, 87 of 2816 genomes sampled between Jan and May 2020 have the T28688C SNV (GISAID, https://nextstrain.org/) that alters the sequence of the binding site of the forward primer of the CDC N3 rRT‐PCR assay,^[^ ^1^ ^]^ potentially compromising its effectiveness. Thus, continued surveillance of the evolution and geographic distribution of viral strains by high‐throughput sequencing^[^ ^3^, ^4^ ^]^ is another pillar of public health measures to combat COVID‐19.

Both rRT‐PCR testing and high‐throughput sequencing of SARS‐CoV‐2 require RNA extraction from nasopharyngeal swab samples. In the clinic, swabs are collected in viral transport media (VTM) and, if necessary, transported following specific cold‐chain biological substances transport guidelines^[^ ^1^ ^]^ for RNA extraction. The US CDC recommends several commercially available RNA extraction kits.^[^ ^1^ ^]^ Fully automated diagnostic systems (e.g., Roche cobas 6800 and 8800) that perform all steps from RNA extraction to rRT‐PCR without human intervention are also popular among diagnostic laboratories. Commercial kits and procedures typically yield consistent quality RNA and are easy to use, but come with a high price tag. Moreover, the availability of commercial proprietary reagents is seriously affected by the disruption of the global supply chain caused by the COVID‐19 pandemic. The high cost and low availability of proprietary reagents impose a bottleneck on testing capacities in rich and poor countries alike. Additionally, since the first reports of SARS‐CoV‐2 shedding in stool,^[^ ^5^, ^6^ ^]^ the presence of the virus has been confirmed in municipal wastewater, sometimes even before the first confirmed cases in the community.^[^ ^7^ ^]^ This suggests that wastewater surveillance is an important public health measure, and it could be effective for monitoring the total COVID‐19 case load (including asymptomatic cases) in the population. Monitoring pathogens in wastewater requires methods that satisfy the biosafety requirements of handling unknown infectious agents and can overcome the low virus concentration and PCR inhibitors that are ubiquitous in wastewater. Therefore, there is great incentive to develop alternative methods that only require locally available and inexpensive chemicals, are simple to perform, and rival the performance of commercial kits. Besides alleviating supply shortage, the alternative methods should ideally eliminate the risk of handling live viruses, thus lowering the strict biosafety and biosecurity requirements^[^ ^8^ ^]^ on testing facilities. Any self‐build RNA extraction method that satisfies the above‐mentioned criteria can help increase testing capacity not only in clinical laboratories but also in rural healthcare facilities, university laboratories and field testing sites.

RNA isolation by acid guanidinium thiocyanate‐phenol‐chloroform extraction (AGPC)^[^ ^9^ ^]^ (sold as TRIzol by Invitrogen or TRI Reagent by Sigma‐Aldrich) has been successfully used in life sciences laboratories around the world for nearly four decades. It requires widely available chemicals at a low cost. Previous studies indicate that TRIzol inactivates several infectious virus,^[^ ^10^ ^]^ including the Middle East Respiratory Syndrome Coronavirus (MERS‐CoV)^[^ ^11^ ^]^ and SARS‐CoV‐2.^[^ ^12^, ^13^, ^14^, ^15^, ^16^ ^]^ In addition, TRIzol reagent had the least effect on the RNA quality.^[^ ^13^, ^17^ ^]^ The AGPC methods has been found to match the performance of commercial kits and automated systems in SARS‐CoV‐2 rRT‐PCR detection.^[^ ^18^, ^19^ ^]^ In these studies, swabs were first collected in VTM or cell culture media, which were then used in AGPC RNA isolation. This workflow necessitates handling of live viruses and requires Biosafety Level 3 (BSL‐3) or BSL‐2 with enhance containment practices (BSL‐2+) facilities.^[^ ^12^, ^20^ ^]^ We hypothesized that it should be possible to collect swabs directly in AGPC, which achieve two goals: 1) complete inactivation of any infectious agent by AGPC so that the downstream procedures (e.g., transportation, RNA isolation, rRT‐PCR, and sequencing) can be performed at relaxed biosafety levels, and 2) preservation of RNA integrity by denaturing nucleases.

However, the AGPC method as is commonly practiced has several drawbacks that make it unsuitable for high‐volume testing. It requires extensive manual pipetting of hazardous chemicals and multiple centrifugation steps, which increase the risk of human errors and personnel injury especially. In the last decade, synthetic magnetic nanoparticles (MNPs) have been developed for various applications.^[^ ^21^, ^22^, ^23^, ^24^, ^25^, ^26^ ^]^ Solid‐phase reversible immobilization (SPRI) of nucleic acid on MNPs offers a simple and elegant alternative to centrifuge‐ or column‐based methods.^[^ ^27^ ^]^ Under dehydrating conditions the MNPs are able to reversibly bind to nucleic acids (e.g., RNA) present in the sample. In the presence of a magnetic field nucleic acids are rapidly separated from most impurities, and the purified nucleic acids can be further released from the surface of MNPs by elution buffer with a different ionic strength.^[^ ^28^, ^29^, ^30^, ^31^ ^]^ SPRI allows fast and thorough washes to eliminate inhibitors of downstream molecular biology reactions and yields high quality RNA for PCR and high‐throughput sequencing. Because it requires no centrifugation and only low‐cost materials, the MNP‐based RNA extraction is inherently scalable and amenable to automation.

In response to the COVID‐19 pandemic, commercial MNP‐based protocols for RNA extraction of COVID‐19 samples have been reported.^[^ ^32^, ^33^, ^34^ ^]^ However, open‐source protocols based on home‐made MNPs are limited. Although some studies suggested that lab‐made MNPs could potentially work for COVID‐19 diagnosis, they suffer from either a lack of efficiency data on COVID‐19 samples^[^ ^35^, ^36^ ^]^ or lower efficacy compared to commercial kits.^[^ ^37^ ^]^ Furthermore, the compatibility of MNPs and AGPC‐inactivated COVID‐19 samples was not tested.^[^ ^30^ ^]^ Although the combination of the AGPC and SPRI technologies would be obviously advantageous in consideration of reagent availability, cost, biosafety and ease‐of‐use, development of AGPC compatible MNP‐based RNA extraction protocols has been limited.

**Figure 1:**
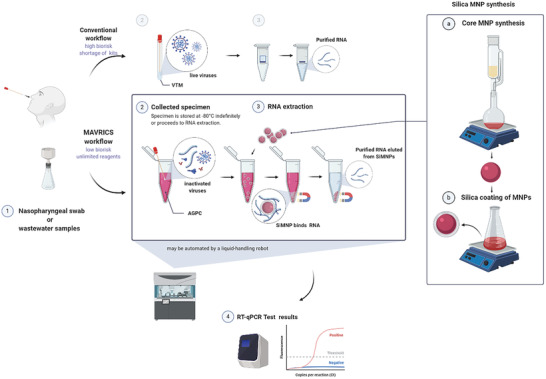
Silica‐coated magnetic nanoparticles can isolate RNA directly from AGPC inactivated samples. A schematic comparison of the conventional and magnetic nanoparticle‐aided viral RNA isolation from contagious samples (MAVRICS) workflow for the detection of SARS‐CoV‐2, influenza viruses, and respiratory syncytial virus in clinical and environmental samples. Figure created with BioRender.com.

Here we developed the magnetic‐nanoparticle‐aided viral RNA isolation from contagious samples (MAVRICS) workflow (**Figure** **1**). MAVRICS only requires widely available and low‐cost materials and can be self‐assembled in a basic laboratory setting. It is compatible with AGPC inactivated samples to alleviate the shortage of commercial kits, lower biosafety risks, and enable sample and scalable sample preparation. MAVRICS performed on par or better than commercial RNA extraction kits in rRT‐PCR detection of SARS‐CoV‐2, influenza viruses and respiratory syncytial virus in clinical and environmental samples.

## 2 Results

### 2.1 Silica‐ But Not Carboxyl‐Coated Magnetic Nanoparticles Can Isolate RNA Directly from AGPC Inactivated Samples

MNPs can be functionalized with either a carboxyl or silica coating to bind nucleic acids.^[^ ^30^ ^]^ Carboxylated MNPs (cMNPs) are available commercially (e.g., RNAClean XP from Beckman Coulter) and widely used in molecular biology workflows such as PCR cleanup and sequencing library preparation. Unfortunately, cMNPs (in the form of RNAClean XP) failed to recover detectable RNA from AGPC solutions (in the form of TRIzol) spiked with high quality total RNA from human cells, while the conventional AGPC method based on organic phase separation and centrifugation recovered ≈45% of input RNA. On the other hand, cMNPs were capable of 96% recovery when the same RNA was spiked in water, suggesting that AGPC interferes with RNA binding onto cMNPs (Table S1, Supporting Information). Silica magnetic nanoparticles (SiMNP) have been used to extract total nucleic acid from samples lysed and inactivated in AGPC without centrifugation and phase separation.^[^ ^30^ ^]^ Since commercial SiMNPs are expensive and difficult to procure during the COVID‐19 crisis, we synthesized SiMNP from scratch using a published open‐source protocol.^[^ ^30^ ^]^ The synthesis took ≈14 h with 3 h hands‐on time and required only base chemicals, a strong magnet, and standard lab equipment (Figure 1A, Figure S1, Supporting Information). In our case, all materials were locally available (**Table** **1**). One synthesis yielded enough SiMNPs for 5000–10 000 extractions, and the material cost was ≈$20 per synthesis, making the average cost per extraction less than 0.3 cents. Another benefit of SiMNP is its chemical inertness. Our SiMNPs have been stored at room temperature for 23 weeks at the time of writing without noticeable change in performance. The size and zeta potential of SiMNP were evaluated and compared with MNPs of a commercial kit (MagBead, ZYMO RESEARCH). While SiMNP had an average diameter of 720 ± 101 nm, and their zeta potential was ‐40.77 ± 2.9 mV, the commercial MNPs were larger (3086 ± 592 nm) and had a smaller zeta potential (‐21 ± 4.8 mV) (Figure S2, Supporting Information). The high zeta potential of SiMNPs indicates successful functionalization and colloidal stability of the synthesized SiMNPs.^[^ ^35^, ^38^ ^]^

**Table 1:** List of materials for SiMNP synthesis, RNA extraction and rRT‐PCR (The vendors and catalog numbers are those used in this study. Alternative sources can also be used)

Reagent	Supplier	Catalog number
Iron (II) chloride tetrahydrate ≥98% (FeCl_2_ ·4 H2O)	VWR Chemicals	13478‐10‐9
Iron (III) chloride, anhydrous, extra pure (FeCl_3_)	Fisher Scientific	10224390
Sodium hydroxide, ≥99% (NaOH)	Sigma Aldrich	306576‐500G
Hydrochloric acid (36.5 to 38.0%)	Fisher Scientific	A144‐500
Ammonia solution (NH_4_OH, 25%)	Fisher Scientific	10642251
Ethanol absolute ≥99.8%	VWR Chemicals	20821.330
Tetraethyl orthosilicate (≥99%) (GC)	Sigma Aldrich	78‐10‐4
2,2‐bis(hydroxymethyl)‐2,2′,2″‐nitrilotriethanol/bis‐tris (C_8_H_19_NO_5_)	Gold Biotechnology	B‐020‐500
Guanidinium chloride/Gu‐HCl (CH_5_N_3_ · HCl)	Fisher Scientific	BP178‐1
Tris base	Promega Corporation	H5135
Tween 20	Sigma Aldrich	P7949
TRIzol reagent	Life Technologies	15596018
SuperScript IV reverse transcriptase	Invitrogen	18090010
RNase OUT	Invitrogen	10777‐019
TaqMan Fast Advanced Master Mix	Invitrogen	4444556
RNase H	New England BioLabs	M0297L
2019‐nCoV Kit	Integrated Device Technology (IDT)	10006605
Influenza/RSV qPCR assay	Integrated Device Technology (IDT)	1079729
Direct‐Zol RNA Miniprep kit	Zymo Research	R2070
QIAamp viral RNA mini kit	Qiagen	52906
ProtoScript II reverse transcriptase	New England BioLabs	M0368

We first tested if SiMNPs could isolate RNA from contrived SARS‐CoV‐2 saliva samples (see Experimental Section) inactivated in AGPC (in the form of TRIzol). As previously reported, SiMNPs were able to isolate RNA directly from TRIzol using the total nucleic acid extraction protocol (hereafter referred to as TNA protocol).^[^ ^30^ ^]^ We used the US CDC 2019‐nCoV rRT‐PCR assay to quantitate the recovery of SARS‐CoV‐2 sequences. In the experience of ours and others,^[^ ^39^ ^]^ there is little difference in the performance of the 2019‐nCoV_N1, N2 or N3 rRT‐PCR assays using contrived SARS‐CoV‐2 samples or COVID‐19 samples (see Experimental Section for a detailed note on the choice of assays in this study). SiMNPs coupled with the TNA protocol resulted an increase of 3.1 in Ct value compared to the official TRIzol Reagent protocol, which means a 11.1% yield of viral RNA relative to the AGPC method (Figure S3A,B, Supporting Information). In contrast, RNA recovered by the cMNP (RNAClean XP) methods was negligible (Figure S3, Supporting Information). Together, these results showed that SiMNPs could isolate viral RNA directly from AGPC inactivated samples, but existing SiMNP protocols significantly underperformed compared to the AGPC method, thus reducing the sensitivity of diagnostic tests.

### 2.2 Development of a SiMNP‐Based Protocol to Maximize Viral RNA Recovery

**Figure 2:**
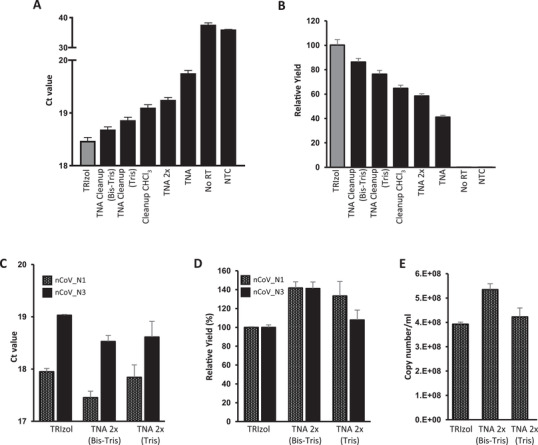
Optimization of SiMNP protocol to maximize viral RNA recovery. A,B) The SARS‐CoV‐2 RNA recovery of various SiMNP protocols was compared using the 2019‐nCoV_N3 rRT‐PCR assay. A) Ct values. B) viral RNA yield relative to TRIzol extraction. C,D) The SARS‐CoV‐2 RNA recovery of the optimized SiMNP protocols was analyzed using the 2019‐nCoV_N1 and N3 rRT‐PCR assays. C) Ct values. D) viral RNA yield relative to TRIzol extraction. The relative yield (in %) is calculated by dividing the RNA yield of the test condition (e.g., TNA 2× bis–tris) by the RNA recovered using TRIzol following manufacturer's recommendations. E) Copy number of SARS‐Cov‐2 RNA in the original sample calculated by the standard curve method. Tris: tris–HCl pH 6.5 buffer. Bis–tris: bis–tris, pH 6.5 buffer, TNA 2×: TNA protocol with an additional TRIzol wash. Data are shown as mean ± SEM of three technical replicates in one PCR assay per sample.

Next, we aimed to develop an efficient SiMNP‐based RNA extraction protocol using the contrived SARS‐CoV‐2 samples and US CDC 2019‐nCoV_N1 and N3 rRT‐PCR assays. Increasing the amount of SiMNPs 2.5 times significantly improved the recovery of both the TNA and cleanup CHCl_3_ protocols. We also noticed an improvement by washing the SiMNPs once with TRIzol and RNA binding buffer (1:1), presumably further removing RNases. Nonetheless, none of these protocols could improve upon the TRIzol Reagent protocol (**Figure** **2**A, B, Figure S4, Supporting Information). Since the cleanup CHCl_3_ protocol had consistently outperformed the TNA protocol, we suspected that the RNA binding buffer^[^ ^30^ ^]^ in the TNA protocol might not be optimal. Indeed, after adding buffering agents (Tris‐HCl or Bis‐Tris, pH6.5) to the RNA binding buffer and increasing its guanidinium chloride concentration to 3 m, the yield of RNA doubled (Figure 2A,B, Figure S4B,C, Supporting Information). It is worth noting that Ct values of the two negative controls (one without reverse transcriptase (No RT) and one without template (NTC)) were stochastically detected and much higher (35–40) than test samples, thus having no meaningful effect on the determination of the relative yield of the experimental conditions (Figure 2A,B).

We combined the modifications, i.e., the additional wash step and new binding buffers, that improved the recovery of viral RNA by SiMNPs and showed that they outperformed the TRIzol reagent protocol as judged by both the N1 and N3 rRT‐PCR assays (TNA 2× bis–tris or tris, Figure 2C,D). The number of SARS‐CoV‐2 RNA molecules captured by the SiMNP‐TNA 2× bis–tris or SiMNP‐TNA 2× tris protocol was estimated by the standard curve method to be very close to the input value (Figure 2E). Similar results were obtained using an independent synthesis of SiMNPs, proving the robustness of the protocols (Figure S4E–G, Supporting Information). Because of the high recovery of SARS‐CoV‐2 viral RNA, we name the method (SiMNP coupled to the TNA 2× bis–tris protocol) magnetic‐nanoparticle‐aided viral RNA isolation from contagious samples (MAVRICS). Using MAVRICS 12 samples can be extracted in parallel in ≈70 min (5.8 min per sample). Our preliminary results show that MAVRICS can be carried out by a Tecan FreedomEVO 200 liquid‐handling robot in a 96‐well format.

### 2.3 Comparing Performance of MAVRICS and Commercial RNA Extraction Kits in SARS‐CoV‐2 rRT‐PCR Diagnostic Panel Using Clinical Samples

**Figure 3:**
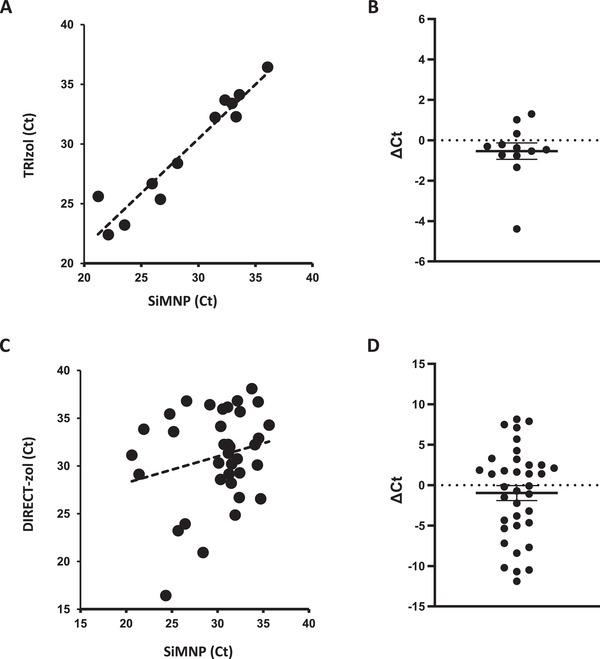
Comparison between MAVRICS and commercial kits in RNA extraction from COVID‐19 clinical samples. RNA extraction was done from 12 samples using MAVRICS and the TRIzol Reagent protocol (A, B, 2019‐nCoV_N3 assay) or 36 samples using MAVRICS and the DIRECT‐zol protocol (C,D, 2019‐nCoV_N1 assay). The graphs show the correlation between A,C) Ct values and B,D) ΔCt values (mean and standard errors are shown).

We next compared MAVRICS with commercial kits using real‐world COVID‐19 swab samples obtained in hospitals in the Western Region of Saudi Arabia. These swabs were directly inactivated in TRIzol at the time of collection. Equal aliquots of 12 COVID‐19 samples lysed in TRIzol (S659‐S670) were extracted using the MAVRICS and TRIzol Reagent protocol respectively. The Ct values (N3 rRT‐PCR assay) obtained by both methods were highly concordant (correlation coefficient = 0.96, **Figure** **3**A). MAVRICS on average provided a reduction in Ct value (0.54 ± 0.41, Figure 3B). We further used these 12 samples and additional 24 samples to compare MAVRICS with the DIRECT‐zol RNA kit, which is a proprietary column‐based method for RNA purification from TRIzol or similar AGPC reagents. The correlation coefficient between the Ct value of MAVRICS and DIRECT‐zol was 0.22 and 0.13, for the US CDC 2019‐nCoV_N1 and N2 rRT‐PCR assays, respectively (Figure 3C and Figure S5A, Supporting Information). Again, MAVRICS on average provided a reduction in Ct value for both N1 and N2 assays (N1: ‐0.98 ± 0.92, N2: ‐0.31 ± 1.0 (Figure 3D, Figure S5B, Supporting Information). The virus load in the 36 samples was estimated to range between 6.84 × 10^3^ and 7.52 × 10^7^ copies per mL (Figure S5C, Supporting information).

### 2.4 MAVRICS Is Compatible with Detection of SARS‐CoV‐2 and Other Viruses in Wastewater Samples

**Figure 4:**
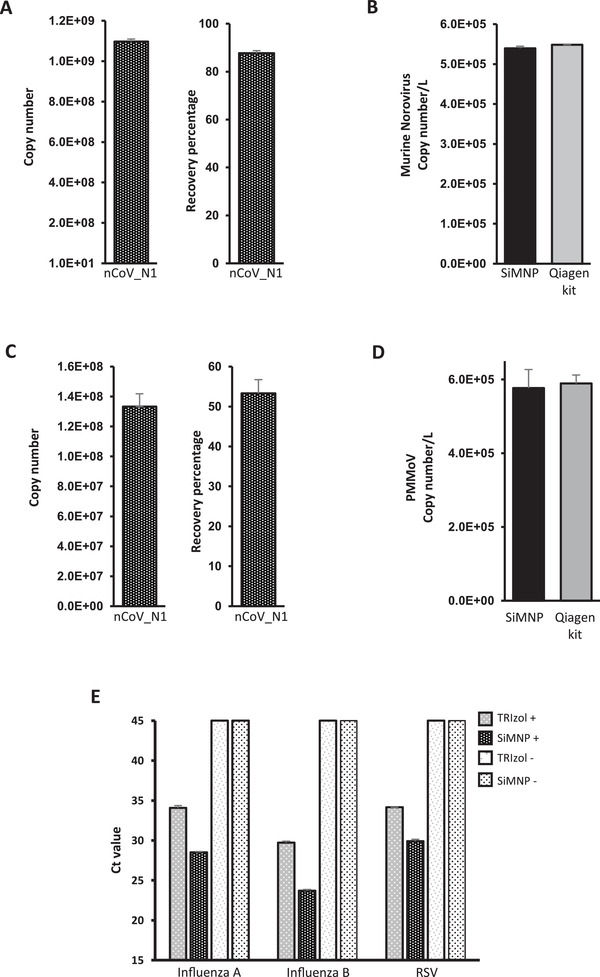
MAVRICS is compatible with wastewater surveillance and detection of other human pathogenic viruses. RNA was extracted by MAVRICS or by the QIAamp viral RNA mini kit (Qiagen kit) from A,B) wastewater concentrate samples spiked with SARS‐CoV‐2 RNA and intact murine noroviruses (MNVs). C,D) wastewater biomass immobilized on electronegative membranes with SARS‐CoV‐2 RNA spike‐in. A, C) the SARS‐CoV‐2 RNA copy numbers were calculated by the standard curve method. The recovery of viral RNA was compared to the input amount. B,D) MNV and PPMoV copy numbers in the original sample were compared between MAVRICS and Qiagen kit. E) RNA was extracted using MAVRICS or the TRIzol reagent protocol from a human respiratory pathogens control panel (influenza A and B viruses, and respiratory syncytial virus (RSV)). The Ct was obtained using a clinical diagnostic rRT‐PCR panel to quantitate the viruses. TRIzol+ and SiMNP+: positive control panel containing pathogens. TRIzol‐ and SiMNP‐: blank control panel without microorganism. Data are shown as mean ± SEM of 3 A,D), 6 B SiMNP), 2 B Qiagen kit), 6 C), or 5 E) technical replicates in one PCR assay per sample.

Since MAVRICS rivaled commercial kit in tests using clinical samples, we hypothesized that it could be a safe and easy‐to‐implement workflow to extract viral RNA in wastewater. We first tested the recovery of known quantities of SARS‐CoV‐2 RNA and intact murine noroviruses (MNVs) spiked in wastewater concentrate, in which viral particles in 250 mL raw sewage were concentrated on electronegative membranes followed by ultrafiltration with Centripep YM‐50 to a final volume of 700 µL.^[^ ^40^ ^]^ The wastewater concentrate was first inactivated by 10× volume of TRIzol and extracted using MAVRICS. The result showed an 88% recovery of the input SARS‐CoV‐2 RNA (**Figure** **4**A). The amount of norovirus RNA captured by the SiMNPs was almost identical to that by the conventional Qiagen RNA purification kit (Figure 4B).

We further simplified the preparation of wastewater by using TRIzol to inactivate and lyse the sewage biomass (including viral particles) immobilized on the electronegative membranes, followed by RNA extraction by MAVRICS. Again, the spike‐in SARS‐CoV‐2 was efficiently recovered (Figure 4C), and the amount of pepper mild mottle virus (PPMoV, ubiquitous in wastewater) captured by the SiMNPs was almost identical to that by the conventional QIAamp viral RNA mini kit (Figure 4D).

### 2.5 MAVRICS Is Compatible with Detection of Influenza A/B and Respiratory Syncytial Virus

Last, we validated the MAVRICS method for detection of other human pathogenic viruses than SARS‐CoV‐2. A commercial human respiratory pathogens control panel that contains influenza A and B viruses, and respiratory syncytial virus (RSV) was lysed in TRIzol and used for RNA extraction by MAVRICS. We then used a clinical diagnostic rRT‐PCR panel to quantitate the viruses. Interestingly, influenza A, influenza B and RSV were readily detectable in samples extracted using SiMNPs, but the Ct value of the same pathogens lagged by 4.08, 6.03, and 5.57, respectively, for samples extracted using the TRIzol Reagent protocol (Figure 4E). No virus was detected in blank controls extracted either by SiMNPs or TRIzol (Figure 4E).

## 3 Discussion

We described a SiMNP‐based RNA extraction workflow, MAVRICS, that is compatible with pathogen detection in clinical and environmental samples. All reagents used in MAVRICS are either readily available or can be synthesized in any biology laboratory with basic equipment. The longest preparation step is the synthesis and silica coating of MNPs, which can be done overnight with ≈3 h hands‐on time. The material cost for one synthesis is inconsequential yet can support thousands of RNA extractions. Because MAVRICS works for samples inactivated and preserved in AGPC (e.g., TRIzol), it allows potentially infectious samples to be handled safely without special biocontainment facilities. Importantly, MAVRICS matches, and often exceeds, the performance of commercial proprietary reagents using established molecular diagnostic tests of SARS‐CoV‐2, influenza viruses, and RSV (Figures 3 and 4B,D,E). These tests entail molecular biology reactions that require high quality input RNA. This suggests that the RNA produced by MAVRICS is free of contaminants and maintains good integrity. It will be of interest to study if MAVRICS is compatible with other molecular biology techniques, such as next‐generation sequencing (NGS), in the future. Since NGS library preparation uses similar reactions, including reverse transcription and PCR, one would expect the answer is affirmative.

We noticed that the correlation between SiMNP and DIRECT‐zol was lower than that between SiMNP and TRIzol (compare Figure 3A,C). In the case of SiMNP versus TRIzol, each sample was divided equally between SiMNP and TRIzol protocols and processed in parallel. On the other hand, the samples used in the SiMNP and DIRECT‐zol comparison was extracted at different times. This was due to clinical reasons. Priority was given to extract enough RNA for NGS using the DIRECT‐zol kits. As a result, samples were not equally divided between the SiMNP and DIRECT‐zol extractions, and the swab might be present in one but not the other extraction method. These reasons could be contributed to the lower correlation between the two methods. Nonetheless, evidence from 36 clinical samples, 2 wastewater samples and 1 pathogens control sample showed that MAVRICS rivals the performance of commercial reagents.

We noticed an interesting lack of correlation between the amount of total RNA and viral RNA (Figure S2A–C, Supporting Information, Figure S4D–G, Supporting information, and Figure S5D, Supporting information vs Figure 3A,B). For example, the RNA concentration of S667 was below the detection range of Qubit fluorometer, and yet the copy number of SARS‐CoV‐2 was higher than S659, which had one of the highest RNA concentrations (Figure S4C,D, Supporting Information). SiMNP tends to have lower total RNA yield, but has lower Ct values when compared to other methods (Figure S4C, Supporting Information). There could be at least two possibilities. First, SiMNPs may favor the binding of RNA similar to the viral RNA. This could be due to the surface chemistry or high surface area to mass ratio of nanoparticles. Second, SiMNPs may be more efficient in removing contaminants that inhibit reverse transcription and PCR. The exact reasons for this phenomenon need to be further studied.

## 4 Conclusion

In this study, we developed an open‐source method for magnetic‐nanoparticle‐aided viral RNA isolation from contagious samples (MAVRICS). This protocol enables safe, economical, and effective extraction of RNA from clinical and environmental samples. Its performance rivals commercial RNA extraction kits in validated diagnostic tests of SARS‐CoV‐2, influenza, viruses, and respiratory syncytial virus. Because this protocol is centrifuge‐free, ongoing and future work will focus on automated high‐throughput of RNA extraction by liquid‐handling robots. In conclusion, MAVRICS has the potential to become an enabling technology for widespread community testing and wastewater monitoring in the current and future pandemics.

## 5 Experimental Section

### Clinical Samples

Contrived SARS‐CoV‐2 saliva samples were prepared by mixing 1000 µL of TRIzol, 100 µL of saliva from a health volunteer, and 5 µL of in vitro transcribed SARS‐CoV‐2 N gene RNA (nt28287‐29230 in NC_045512.2, 10^8^ copies per µL). Anonymized RNA samples were obtained from the Ministry of Health (MOH) hospitals in the western region in Saudi Arabia. The use of clinical samples in this study is approved by the institutional review board (IRB# H‐02‐K‐076‐0320‐279) of MOH and KAUST Institutional Biosafety and Bioethics Committee (IBEC). Oropharyngeal and nasopharyngeal swabs were carried out by physicians and samples were steeped in 1 mL of TRIzol (Invitrogen Cat. No 15596018) to inactivate the virus during transportation. The respiratory (21 targets) control panel (Microbiologics Cat. No 8217) was used as controls in rRT‐PCR assays.

### Wastewater Samples and Virus Concentration

One liter of raw sewage was individually sampled at 9 AM and 4 PM on 7 June 2020 from the equalization tank of wastewater treatment plant operated within KAUST. The sewage from both time‐points was then mixed together to constitute a composite sample. Raw sewage (300–500 mL) was concentrated by using an electronegative membrane in the presence of cation which was described previously.^[^ ^40^ ^]^ Briefly, 2.5 m MgCl_2_ was added to the water samples to obtain a final concentration of 25 × 10^‐3^ m. The samples were subsequently passed through the electronegative filter (cat. no. HAWP‐090‐00; Merck Millipore, Billerica, MA) attached to a glass filter holder (Merck Millipore, Cat no. XX1009020). Magnesium ions were removed by passing 200 mL of 0.5 × 10^‐3^ m H_2_SO_4_ (pH 3.0) through the filter, and the viruses were eluted with 10 mL of 1.0 × 10^‐3^ m NaOH (pH 10.8). The eluate was recovered in a tube containing 50 µL of 100 × 10^‐3^ m H2SO4 (pH 1.0) and 100 µL of 100 × tris–EDTA buffer (pH 8.0) for neutralization. The samples were further concentrated using a Centripep YM‐50 (Merck Millipore) to obtain a final volume of 600–700 µL.

### Magnetic Nanoparticle Synthesis, Silica Coating and RNA Extraction Protocol

Core magnetic nanoparticle synthesis and silica coating of MNPs were done following published protocols (Protocols 1.1 and 2.1 in reference 29). A detailed supplementary protocol 1 can be found in online Supporting Information. A step‐by‐step protocol is also available at url: https://doi.org/10.17504/protocols.io.bik4kcyw.

### Size and Zeta Potential Evaluation

Size and zeta potential measurements, in SiMNPs and in commercial magnetic beads (ZIMO RESEARCH, Direct‐zol Cat num. R2102), were obtained using a Zetasizer nano Series (Malvern). Prior to measurements the sample concentrations were adjusted to 0.1% w/v particles in ddH_2_O, and sonicated (Ultrasonic cleaner JSP US21) for 1 min.

### RNA Extraction by Commercial Methods

RNA extraction was performed using the Direct‐Zol RNA Miniprep kit (Zymo Research Cat. No R2070), TRIzol reagent (Invitrogen Cat. No 15596026), or RNAClean XP beads following the manufacturer instructions. Viral RNA was extracted from the concentrated raw sewage by using QIAamp viral RNA mini kit (Qiagen, cat no: 52906) following manufacture instruction. A 140 µL of concentrated raw sewage was used to obtain a final elution of 80 µL. The RNA was stored in ‐20 °C freezer until further use.

### Reverse Transcription

Reverse transcription of RNA samples was done using either NEB ProtoScript II reverse transcriptase (NEB Cat. No M0368) or Invitrogen SuperScript IV reverse transcriptase (Thermo Fisher Scientific Cat. No 18090010), following protocols provided by the manufacturers. After reverse transcription, 5 units of RNase H (New England Biolabs Cat. No M0523S) was added and incubated at 37 °C for 20 min to remove RNA. All of the web‐lab experiments in this study were conducted in a horizontal flow clean bench to prevent contaminations. The bench was decontaminated with 70% ethanol, DNA*Zap* (Invitrogen, Cat no. AM9890) and RNase *AWAY* (Invitrogen, Cat no. 10328011) before and after use. The filtered pipette tips (Eppendorf epT.I.P.S. LoRetention series) and centrifuge tubes (Eppendorf DNA LoBind Tubes, Cat. No 0030108051) used in this study were PCR‐clean grade.

### Real‐Time PCR

Real‐time PCR assays for SARS‐CoV‐2 were purchased from IDT (Cat. No 10006770). Real‐time PCR analysis of SARS‐CoV‐2 sequences was analyzed on a CFX384 touch real‐time PCR detection system (Bio‐rad) using the following program: 50 °C for 2 min, 95 °C for 2 min followed by 45 cycles of 95 °C for 5 s and 59 °C for 30 s. Real‐time PCR assays for influenza A, B/RSV were purchase from IDT (Cat. No 1079729) and used per manufacturer recommendation. For influenza and RSV assays, the following program was used: 50 °C for 2 min, 55 °C for 120 s, 60 °C for 360 s, 65 °C for 240 s, followed by five cycles 95 °C for 5 s and 55 °C for 30 s, and then 45 cycles of 91 °C for 5 s and 58 °C for 25 s. MNV and PMMoV real‐time PCR assay was conducted using the primer and probes, which described previously.^[^ ^41^, ^42^ ^]^ Please note that in the experience of ours and other (ref. ^[^ ^39^ ^]^) the performance of the N1, N2, and N3 assay is similar, and the correlation of Ct value of the three assays in standard curves of SARS‐CoV‐2 samples is high. This indicates that reliable results can be obtained with any of them. When this study was conducted between March and June 2020, Saudi Arabia (where one are located) imposed a ban on international flights. One had limited supplies of real‐time PCR reagents, and it was not clear if one could obtain more in the near future. Because of the comparable performance of the three assays, one decided to use one assay (N3) only in the preliminary optimization phase and save N1 and N2 probes for clinical diagnostic purposes. Thus, the first 12 clinical samples were tested with the N3 assay, and it was done consistently in all figures. Two assays (N1 and N3) were used in the next standardization phase (Figure 2 and Figure S3 Supporting Information), and the objective was to run an independent rRT‐PCR assay other than N3 for each sample to show that the result was reproduced with different probes. Later, when more reagents were secured, N1 and N2 assays were performed as CDC recommends for all COVID‐19 samples (Figure 3 and Figure S4 Supporting Information). Because these clinical samples are very valuable and in limited supply and CDC no longer recommends the N3 probe for diagnosis, one did not repeat N3 for the later 24 samples. Regardless of the probe used, the performance of the SiMNPs remained consistent, and therefore the different probes used in the two batches of samples did not affect the conclusions of the manuscript.

## Conflict of Interest

The authors declare no conflict of interest.

## Author Contributions

M.L. and G.R.M. performed majority of the molecular biology experiments. M.L. and G.R.M. analyzed the data and wrote the manuscript. R.S., S.M., and J.X. performed experiments. A.R. and P.H. performed experiments on wastewater. DBS performed the evaluations of size and zeta potential. FSA, AK, AMH, NAMA and AA collected clinical samples. SH and AP coordinated the clinical samples and molecular testing. ML conceived and supervised the study.

## Supporting information

Supporting Information

## Data Availability

The data that support the findings of this study are available from the corresponding author upon reasonable request.
