# Supplementary material for: A Robust, Safe, and Scalable Magnetic Nanoparticle Workflow for RNA Extraction of Pathogens from Clinical and Wastewater Samples
Source: Glob Chall. 2021 Feb 22;5(4):2000068. doi: 10.1002/gch2.202000068 (PMC7995109; doi:10.1002/gch2.202000068)
Supplement: Supplementary file 1 — Supporting Information [file GCH2-5-2000068-s001.pdf]

© 2021 Wiley-VCH GmbH

Global Challenges
Open Access

# Supporting Information

for *Global Challenges,* DOI: 10.1002/gch2.202000068

# A Robust, Safe, and Scalable Magnetic Nanoparticle Workflow for RNA Extraction of Pathogens from Clinical and Wastewater Samples

*Gerardo Ramos-Mandujano, Rahul Salunke, Sara Mfarrej, Andri Taruna Rachmadi, Sharif Hala, Jinna Xu, Fadwa S. Alofi, Asim Khogeer, Anwar M. Hashem, Naif A. M. Almontashiri, Afrah Alsomali, Digambar B. Shinde, Samir Hamdan, Pei-Ying Hong, Arnab Pain, and Mo Li**

## Supporting Information

# A Robust, Safe and Scalable Magnetic Nanoparticle Workflow for RNA Extraction of Pathogens from Clinical and Wastewater Samples

**Authors:** Gerardo Ramos-Mandujano, Rahul Salunke, Sara Mfarrej, Andri Rachmadi, Sharif Hala, Jinna Xu, Fadwa S. Alofi, Asim Khogeer, Anwar M. Hashem, Naif A.M. Almontashiri, Afrah Alsomali, Digambar B. Shinde, Samir Hamdan, Peiying Hong, Arnab Pain, Mo Li*

**Supporting information: Figure S1-S5**

**Table 1 Supporting Information**

**Supplementary protocol 1**

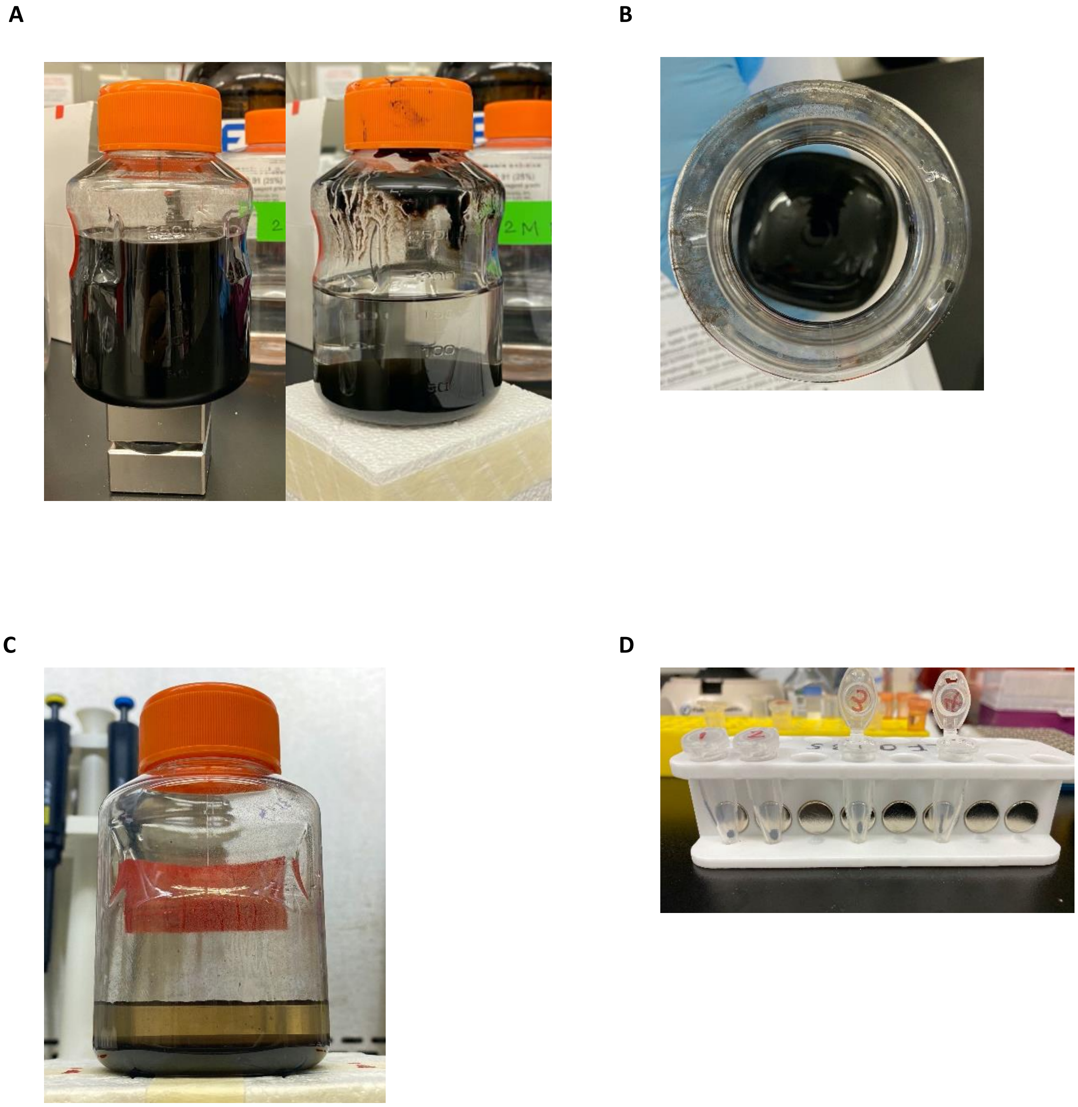


**Figure S1 Supporting Information .** SiMNP synthesis. **A.** Pictures of the synthesized MNPs before (left) and after (right) pelleting by a strong magnet. **B.** A picture of the MNP pellet after decanting the solution. **C.** SiMNPs stored in equal volume of RNase-free water. **D.** A picture of a simple magnetic tube rack used to pellet the SiMNPs in Eppendorf tubes.

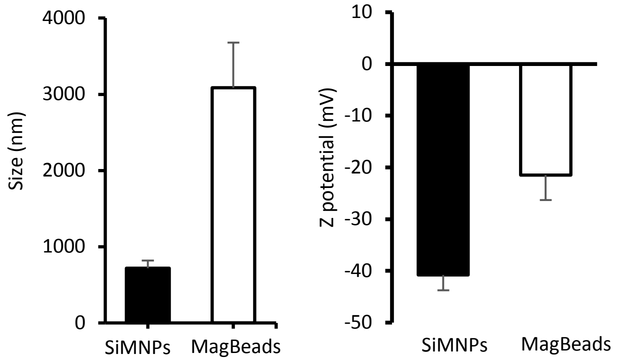


**Figure S2 Supporting Information.** Size and zeta potential determination in SiMNPs and MagBeads. Size (left) and zeta potential (right) measurements were obtained by a Zetasizer nano ZSP (Malvern). Prior to measurements the sample concentrations were adjusted to 0.1% w/v particles in ddH2O, and sonicated (Ultrasonic cleaner JSP US21) for 1 min. n=3-7 independent evaluations, error bars represent standard errors.

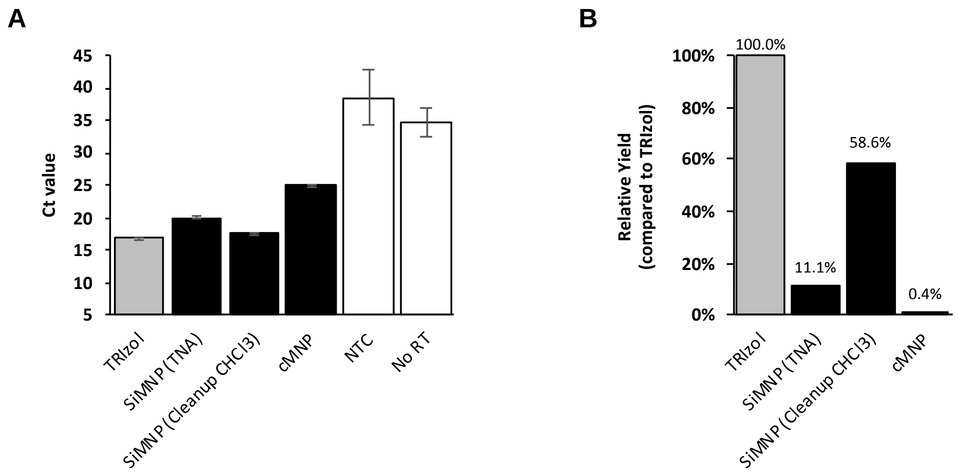


**Figure S3 Supporting Information**. Silica magnetic nanoparticles (SiMNP) synthesized using an open-source protocol are able to isolate viral RNA. RNA was extracted from contrived SARS-CoV-2 saliva samples inactivated in TRIzol using different methods. The TRIzol Reagent protocol was used as a control. After RNA extraction rRT-PCR was conducted with the US CDC 2019-nCoV_N3 assay. **A.** Ct values, and **B.** Viral RNA yield relative to TRIzol extraction. AGPC: acid guanidinium thiocyanate-phenol-chloroform. Interestingly, the yield of SiMNPs improved when the sample in TRIzol was first phase separated by chloroform and the aqueous phase was used in combination with an enzymatic reaction cleanup protocol described in[18](cleanup CHCl3 protocol). However, this modification defeated the purpose of using SiMNPs to simplify the workflow. TNA: total nucleic acid extraction protocol, cMNP: carboxylated MNPs, NTC: no template control, No RT: no reverse transcriptase control. Data are shown as mean ± SEM of three technical replicates in one PCR assay per sample.

A

| PROTOCOL | RNA [ng/µl] |
|---|---|
| TRIzol | 1.16 |
| TNA cleanup (Bis-Tris) | 0.46 |
| TNA cleanup (Tris) | 0.49 |
| Cleanup $CHCl_3$ | ND |
| TNA (2x TRIzol) | 0.54 |
| TNA | 0.74 |

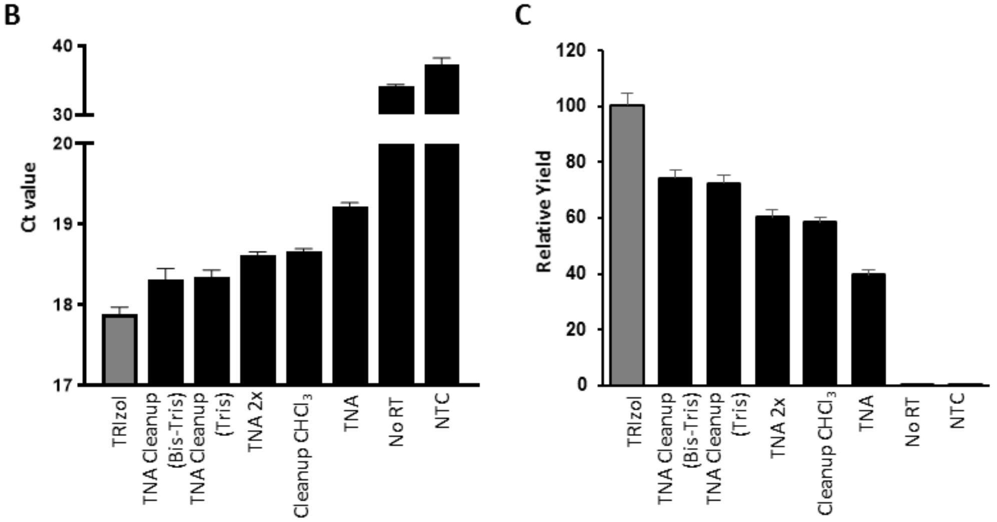


D

| SiMNP 2nd BATCH | RNA [ng/µl] |
|---|---|
| TRIzol | 1.02 |
| TNA 2x (Tris) | 0.72 |

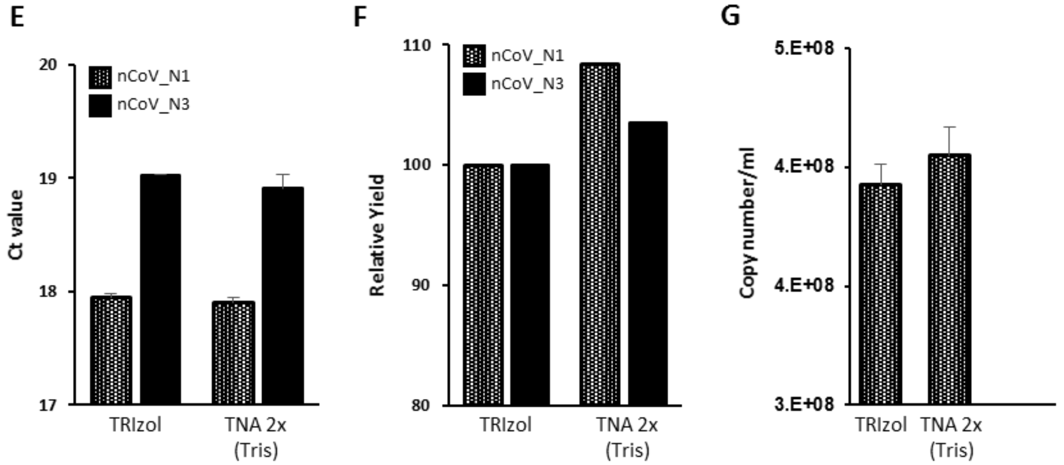


**Figure S4 Supporting Information**. Optimization of SiMNP protocol to improve viral RNA extraction. After extraction using TRIzol or different SiMNP protocols RNA was quantified (**A**). **B-C**: the SARS-CoV-2 RNA recovery was compared using the 2019-nCoV_N1 probe rRT-PCR assays (**B:** Ct values, **C**: viral RNA yield relative to TRIzol extraction). **D-F**: Similar to **A-C** but using a second synthesis of SiMNPs. In the experiment using the second batch of SiMNP, the copy number of SARS-Cov-2 RNA in the original sample calculated by the standard curve method (**G**). Tris: Tris-HCl pH6.5 buffer. Bis-Tris: Bis-Tris, pH6.5 buffer, TNA 2x: TNA

protocol with an additional TRIzol wash. Data are shown as mean ± SEM  of three technical replicates in one PCR assay per sample.

A

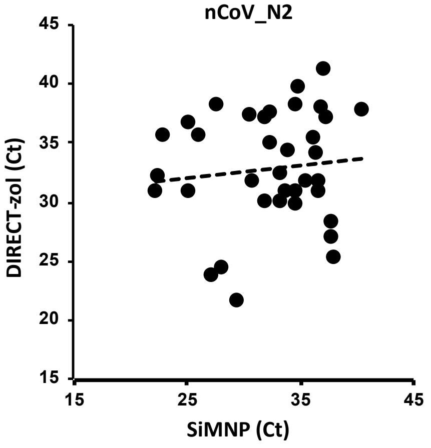


B

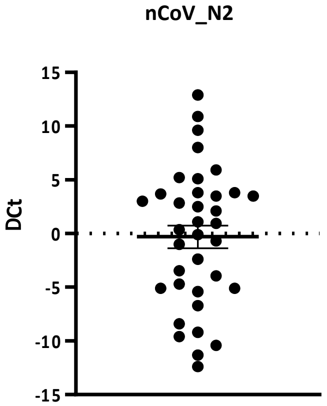


C

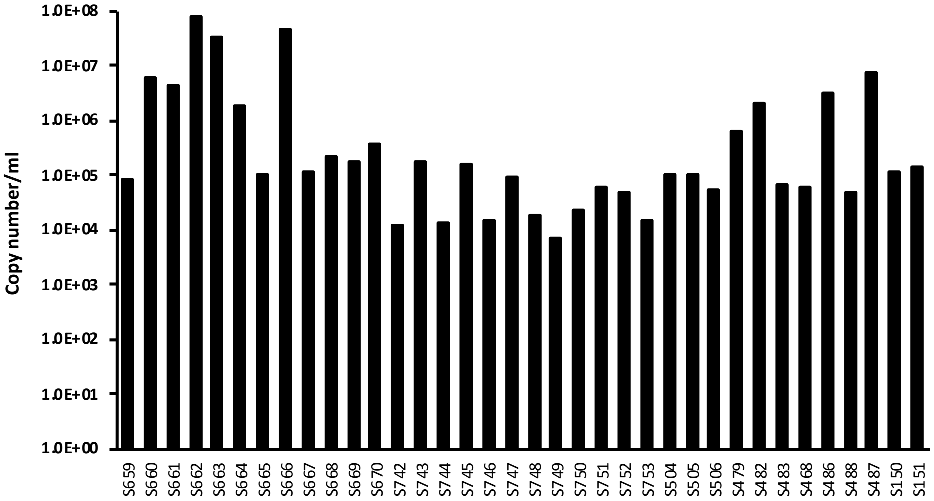


D

| | RNA [ng/µl] | |
|---|---|---|
| **SAMPLE** | **SiMNP** | **TRIzol** |
| **S659** | 21.3 | 78.6 |
| **S660** | 8.5 | 20.6 |
| **S661** | 27.1 | 77.4 |
| **S662** | 9.1 | 17.8 |
| **S663** | 15.9 | 74.1 |
| **S664** | 22.5 | 69.1 |
| **S665** | 4.2 | 8.5 |
| **S666** | 9.7 | 29.9 |
| **S667** | ND | ND |
| **S668** | 32.9 | 64.0 |
| **S669** | 4.5 | 15.9 |
| **S670** | 30.1 | 34.5 |

**Figure S5 Supporting Information** . RNA extraction from COVID-19 clinical samples and comparison between MAVRICS and commercial kits. **A-B**. RNA extraction was done from 36 samples using MAVRICS or DIRECT-zol protocol and the SARS-CoV-2 RNA recovery was compared using the 2019-nCoV_N2 probe. The graphs show the correlation between Ct values (**A**) and ∆Ct value (**B**, mean and standard errors are shown). **C**. SARS-CoV-2 viral load (Copy number/ml) of 36 samples obtained using the MAVRICS protocol. **D**. Total RNA yield by SiMNP and TRIzol protocol. RNA concentration was evaluated by the Qubit fluorometer method.

**Table 1 Supporting Information.** Evaluation of RNA extraction performance of cMNPs in AGPC solutions

| Reagent | Extraction method | Sample buffer | Input RNA (ng) | RNA after extraction by Qubit (ng) | Yield |
|---|---|---|---|---|---|
| **RNAclean XP beads** | RNAclean XP protocol | TRIzol | 141.7 | Too low | ND |
| **RNAclean XP beads** | RNAclean XP protocol | 50%TRIzol, 50% H2O | 141.7 | Too low | ND |
| **RNAclean XP beads** | RNAclean XP protocol | 50% TRIzol, 50% EtOH | 141.7 | Too low | ND |
| **RNAclean XP beads** | RNAclean XP protocol | H2O | 141.7 | 136.2 | 96% |
| **Trizol** | TRIzol Reagent | TRIzol | 141.7 | 63.6 | 45% |

ND: not determined

**Supplementary protocol 1. Magnetic nanoparticle synthesis, silica coating and RNA extraction.**

Silica magnetic nanoparticles (SiMNP) synthesis.

1. Core magnetic nanoparticle synthesis and silica coating of MNPs were done following published protocols (Protocols 1.1 and 2.1 in reference 12).

Preparation of Bis-Tris Buffer (50 mL)

1. Dissolve 14.33 g guanidinium hydrochloride and 104.6 mg Bis-Tris in 45 mL of 100% ethanol. NOTE: Add 40 ml of 100% ethanol to the other chemicals, and wait for guanidinium hydrochloride to completely dissolve and add the remaining volume of 100% ethanol.

2. Adjust pH (<6.5) with HCl, and adjust the volume with $H_2O$ to 50 mL.

Sample preparation.

1. Oropharyngeal or nasopharyngeal swabs were steeped in 1 mL acid guanidinium thiocyanate-phenol-chloroform (AGPC, e.g., TRIzol Reagent or TRI reagent).

RNA extraction

1. In an Eppendorf tube, add 200 µl clinical sample and 200 µl Bis-Tris buffer, mix well by vortexing.

2. Add 40 µl SiMNP, mix 5 min at 1300 rpm.

3. Spin the tube for 2-3 seconds, settle the SiMNPs on a magnetic stand and remove the supernatant. Remove the tube from the magnetic stand.

4. Mix 200 µl of AGPC (TRIzol or TRI reagent) and 200 µl Bis-Tris buffer, add to the SiMNPs, mix well by vortexing.

5. Settle the SiMNPs on a magnetic stand and remove the supernatant. Remove the tube from the magnetic stand.

6. Add 400 µl of 90% ethanol, spin for 2-3 seconds, settle the SiMNPs on a magnetic stand and remove the supernatant. Remove the tube from the magnetic stand.

7. Repeat Step 6 three more times for a total of 4 ethanol washes.

8. After removing the supernatant from the last ethanol wash, dry the SiMNPs on a heat block at 50°C. Keep the lid open, no shaking. Do not elute before the SiMNPs are dried.

9. To elute the RNA, add 40 µl nuclease-free water, and mix 5 min at 1300 rpm at room temperature.

10. Settle the SiMNPs on a magnetic stand and transfer the eluted RNA to a new RNase-free tube.

11. Analyze RNA concentration and purity using a Qubit fluorometer or Nanodrop.

12. Store at -80°C or use immediately.

Reverse transcription (RT) and Real-time PCR

1. RT: use 4 µl of eluted RNA and follow the instructions for SuperScript™ IV Reverse Transcriptase adding the RNase H incubation step.

2. Real-time PCR: For each 10 µl qPCR reaction mix 1.5 µl cDNA, 0.5 µl SARS-CoV-2 (2019-nCoV) CDC qPCR Probe Assay, 5 µl TaqMan Fast Advanced Master Mix, and 1.5 µl nuclease-free water. Run qPCR on a Biorad CFX384 Touch Real-Time PCR Detection System (or similar instrument) using the following program: 50°C for 2 min, 95°C for 2 min followed by 45 cycles of 95°C for 5 sec and 59°C for 30 sec.
